# Opportunistic Infections in Patients with HTLV-1 Infection

**DOI:** 10.1155/2015/943867

**Published:** 2015-11-26

**Authors:** Toshiki Tanaka, Toshio Sekioka, Masakatsu Usui, Shinsaku Imashuku

**Affiliations:** ^1^Department of Internal Medicine, Uji-Tokushukai Medical Center, Uji 611-0042, Japan; ^2^Department of Laboratory Medicine, Uji-Tokushukai Medical Center, Uji 611-0042, Japan

## Abstract

As an acquired immunodeficiency, human immunodeficiency virus (HIV) infection is primarily responsible for opportunistic infections in infected patients. However, opportunistic infections also occur in individuals with human T cell lymphotrophic virus type 1 (HTLV-1) infection. Here, we report opportunistic infections in two Japanese HTLV-1-seropositive patients. The first patient was a 67-year-old male, who had cytomegalovirus infection associated with esophagogastritis and terminal ileitis. The patient was HTLV-1-positive and was diagnosed with smoldering adult T cell leukemia (ATL). High levels of serum soluble IL-2 receptor (sIL-2R; 4,304 U/mL) and an increased percentage of CD4+CD25+ T cells (75.5%) in peripheral blood were also detected. The second patient was a 78-year-old female, a known asymptomatic HTLV-1 carrier, who presented with persistent herpes zoster, followed by *Pneumocystis jirovecii* pneumonia. Disease progression of smoldering ATL along opportunistic infections was observed with very high levels of serum sIL-2R (14,058 U/mL) and an increased percentage of CD4+CD25+ T cells (87.2%) in peripheral blood. In patients with suspected opportunistic infections, both HTLV-1 and HIV should be considered. In HTLV-1-positive patients, an increase in the CD4+CD25+ T cell subset may have its value as a prognostic marker.

## 1. Introduction

Human T cell leukemia virus type 1 (HTLV-1) infection is associated with asymptomatic HTLV-1 carriers and with the development of inflammatory HTLV-1-associated myelopathy/tropical spastic paraparesis (HAM/TSP) or adult T cell leukemia (ATL). Furthermore, HTLV-1 is associated with the development of opportunistic infections [1–4]. The risk of asymptomatic HTLV-1 carriers developing ATL is between 1% and 4% [2], although the factors promoting evolution from the healthy carrier state to symptomatic ATL are not well known. The incidence of opportunistic infections is higher in HTLV-1-infected individuals [3, 4]. As an acquired immunodeficiency, human immunodeficiency virus (HIV) infection is primarily responsible for opportunistic infections in affected patients. Although HTLV-1-infected individuals are not as globally widespread as HIV-infected individuals, a high prevalence of HTLV-1 infection is observed in several countries, including Japan. In such countries, the physician should consider the patients current HTLV-1 infection status when opportunistic infections are suspected. It is believed that such opportunistic infections in HTLV-1-positive patients are not caused by the virus itself, but by alterations in the host's immune functions [1]. In particular, retroviral infection of CD4+CD25+ T cells plays a major role in the pathogenesis of HTLV-1-related immunocompromised status [5–7]. Opportunistic infections in HTLV-1-infected individuals may cause multiple organ dysfunctions due to a mixture of cells responding to infection and infiltration by HTLV-1-infected cells [8]. Management of HTLV-1-associated opportunistic infections is difficult due to the lack of effective antiretroviral agents; thus, patients often have a dismal outcome. Here, we report two HTLV-1-positive elderly patients in Japan, both of whom developed opportunistic infections. In one patient, initial symptoms associated with cytomegalovirus (CMV) gastroenteritis were observed, followed by confirmation of HTLV-1-positive status. The second patient was an identified HTLV-1 carrier who developed persistent herpes zoster and* Pneumocystis jirovecii* pneumonia (PCP). Both patients showed significant increases in the CD4+CD25+ T cell subpopulation in peripheral blood in addition to opportunistic infections.

## 2. Case Report

### 2.1. Case 1

**Figure 1:**
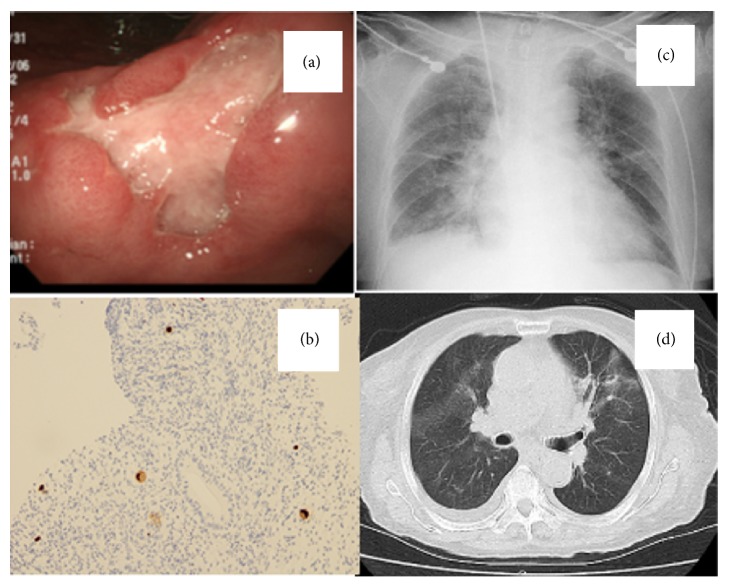
The opportunistic infections in the two HTLV-1-positive patients. (a) Gastric ulcers. (b) Positive staining of cytomegalovirus in a stomach biopsy (original magnification, ×400), from case 1. (c) Chest X-ray and (d) chest computed tomography images of the lungs showing fine nodular shadows and ground glass opacity, from case 2.

**Figure 2:**
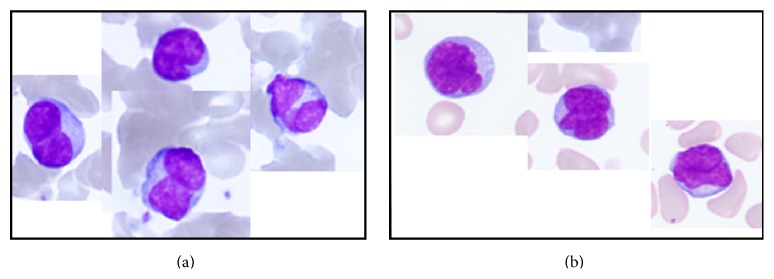
Lymphocytes with atypical convoluted nuclei: (a) in case 1 and (b) in case 2 (May-Grünwald-Giemsa stain; original magnification, ×1000).

In November 2014, a 67-year-old male (height, 163 cm; weight, 44 kg) presented with epigastralgia, anorexia, and excessive weight loss (10 kg per month). Endoscopic studies revealed multiple small ulcers extending from the cardiac to the pyloric region and an irregular shaped ulcer in the angular region of the stomach; however, a biopsy was not performed and the patient was treated with a histamine H2-receptor antagonist. Two months later, the patient presented at our clinic with a high fever, epigastralgia, diarrhea, and continuous anorexia. On admission, the patient was neither anemic nor icteric. No lymphadenopathy was noted. The blood test results are summarized in Table 1. A second endoscopy of the upper gastrointestinal tract revealed several ulcers at the esophagogastric junction of the lower esophagus. The gastric mucosa was edematous and reddish, and multiple ulcers were found in the corpus, angular region and in the greater curvature of the stomach. These results indicated an increase in both the number and size of the ulcers. Biopsies from these lesions showed inflammatory cell infiltrates associated with typical CMV intranuclear inclusion bodies, suggesting the presence of esophagogastric CMV infection (Figures 1(a) and 1(b)). We found it interesting that the patient was negative for CMV-IgM but was positive for CMV antigenemia. Colonoscopy identified two small ulcers in the terminal ileum and a large ulcer (3 cm) in the ileocecal valve. Histopathology of the biopsied tissues revealed CMV infection in the ileum and cecum (data not shown). Although the patient was initially suspected of having Behçet's disease, he was screened for immunodeficiency due to the opportunistic CMV gastroenteritis. The results revealed that the patient was HTLV-1-positive, with a high titer of anti-HTLV-1 antibodies (1 : 1024). He was HIV-negative. A blood smear was carefully reexamined and abnormal lymphocytes with bizarre, convoluted nuclei were identified, although these were not the flower cells typically observed in cases of HTLV-1 infection (Figure 2(a)) [9]. An extremely high CD4+CD25+ T cell count in peripheral blood was identified by flow cytometry (Table 2). The patient was finally diagnosed with smoldering ATL. Initial treatment for Behçet's disease (mesalazine, 1.0 g, 3 times per day) and infliximab (one 300 mg infusion) was changed to ganciclovir (250 mg, twice per day for 14 days) to treat CMV gastroenteritis, which significantly improved CMV antigenemia. However, high fever continued to be unresponsive to prednisolone (maximum 30 mg/day) and intravenous immunoglobulin (5 g/day for 5 days). The patient was transferred to a university hospital for further care.

### 2.2. Case 2

A 78-year-old female (height, 142 cm; weight, 41.8 kg) had tested positive for HTLV-1 with a high titer of anti-HTLV-1 antibodies of 1 : 4,096 four years earlier. During the next 4 years, the patient remained asymptomatic for HTLV-1 infection and did not undergo any specific treatment. In December 2014, the patient was hospitalized with herpes zoster in the left flank region. Laboratory data from 2010 and 2014 are shown in Table 1. The herpes zoster infection resolved following an acyclovir infusion (250 mg, twice per day for 5 days) but reappeared in the left encephalotrigeminal region and spread to the right chest and buttocks over a 3-month period. The patient showed bizarre-looking lymphocytes in the peripheral blood smear (Figure 2(b)). In January 2015, the patient also developed PCP, which was diagnosed on the basis of imaging studies (Figures 1(c) and 1(d)) and the detection of* Pneumocystis jirovecii* (9 × 10^5^/*μ*g DNA) in bronchoalveolar lavage fluid by quantitative polymerase chain reaction. She also had elevated levels of serum *β*-D-glucan (>1,000 pg/mL) (Table 1). The pneumonia resolved following the oral administration of trimethoprim-sulfamethoxazole (TMP-SMX, three tablets, three times per day for 2 weeks). However, in spite of the coadministration of MEPM, VCM, and MCFG, the fever persisted. The patient also had a mass in the right hepatic lobe and subcutaneous masses in the back and perineum; these were suspected to be noninfectious ATL-associated localized lymphomatous lesions [10]. The extremely high levels of serum soluble interleukin-2 receptor (sIL-2R, 14,058 U/mL) and an increase in the percentage of CD4+CD25+ T cells (87.2%) (Table 2) led us to believe that her HTLV-1 infection had progressed from carrier status to smoldering ATL. The patient was hospitalized for 4 months during which time her condition rapidly deteriorated and she died.

## 3. Discussion

Although HTLV-1 carriers do not necessarily develop opportunistic infections [9], Tashiro et al. [11] summarized HTLV-1-related opportunistic infections occurring in 5 carriers and in 6 smoldering ATL cases. In patients with smoldering ATL, it is emphasized that close observation with careful monitoring for opportunistic infections is recommended [12]. In our case 1, an initial diagnosis of CMV gastroenteritis was made, followed by the demonstration of HTLV-1 positivity. However, case 2 was a known HTLV-1 carrier and developed recurrent and intractable herpes zoster and PCP. We did not observe any neurological signs suggestive of HAM/TSP in either of our cases. Upon diagnosis of opportunistic infections, the patients were no longer HTLV-1 healthy carriers; they were in a progressive stage of smoldering ATL. The 3 cases described by Tashiro et al. [11] all developed overt ATL 14–16 months after the onset of opportunistic infections. D'Incan and colleagues [2] also proposed that opportunistic infections, such as the reactivation of varicella-zoster, may promote the development of ATL in healthy HTLV-1 carriers. The progression of asymptomatic HTLV-1 infection to smoldering ATL must be considered when opportunistic infections occur in HTLV-1-positive patients. With regard to these issues, we propose two important diagnostic measures: the first involves screening blood smears for abnormal lymphocytes with bizarre, convoluted nuclei (Figures 2(a) and 2(b)) whereas the second involves monitoring serum sIL-2R levels and the percentage of CD4+CD25+ T cells over time (Table 2). In particular, the latter is quite a useful marker for predicting whether HTLV-1 carriers have developed immunodeficiencies, which may allow opportunistic infections or smoldering ATL to develop. Although both of our cases lacked the clinical signs of acute onset overt ATL, such as lymphadenopathy, hepatosplenomegaly, and hypercalcemia, the opportunistic infections were intractable and their outcome was rather severe because the HTLV-1 infection status was thought to be progressing significantly during the opportunistic infections.

HTLV-1-related opportunistic infections are widely recognized; indeed, CMV gastritis, gastroenterocolitis [13, 14], PCP [15], and disseminated herpes zoster [16] have all been reported. There have also been reports of strongyloidiasis [17], pulmonary histoplasmosis [18], and cryptococcal meningitis [19]. Here, we found that serum *β*-D-glucan levels in case 2 reflected PCP activity; this is consistent with the findings of previous reports [20, 21].

The precise mechanisms underlying the immunocompromised status in HTLV-1-infected individuals remain unknown. Yasunaga and coworkers [4] suggested that suppressed production of T lymphocytes in the thymus may be responsible for the immunodeficiency observed in HTLV-1-infected individuals. A predominance of HTLV-1-infected CD4+ T cells is a well-recognized phenomenon in HTLV-1 carriers; indeed, because HTLV-1 preferentially infects CD4+CD25+ regulatory T cells, thus, CD4+ CD25+ T cells are a major reservoir of HTLV-1 provirus [5, 22]. This may promote inflammation and account for the pathobiology of HTLV-1 transformation and ATL development [23]. The profound immunosuppression observed in HTLV-1-infected individuals is thought to be associated with an increase in the CD4+CD25+ suppressor phenotype. These regulatory T cell counts were described to increase in association with susceptibility to* Strongyloides* hyperinfection [24]. Furthermore, Foxp3 expression by CD4+CD25+Foxp3+ regulatory T cells is lower in HTLV-1-infected individuals than in healthy controls [6, 7]. In both of our patients, the regulatory CD4+CD25+ T cell subset was significantly increased when opportunistic infections occurred, although we did not examine Foxp3 expression. In our observation, monitoring the CD4+CD25+ T cell subset over time in the occurrence of opportunistic infections seems to be a useful prognostic biomarker in HTLV-1 carriers and in smoldering ATL.

Treatment of HTLV-1-associated opportunistic infections is rather difficult due to the immunodeficient status of the patient and the infiltration of various organs by HTLV-1-infected cells. Antiviral agents such as acyclovir and ganciclovir alone do not cure the disease. Aggressive chemotherapy may be required to kill HTLV-1-infected CD4+ cells, but increasing the risk of opportunistic infections and further immunosuppression. The cases described herein were not candidates for allogeneic hematopoietic stem cell transplantation (allo-HSCT) due to their age. We attempted to treat case 2 with etoposide [25]; however, its effectiveness was limited. A recent study in Japan reported that administration of the anti-CCR4 antibody, mogamulizumab, was effective and resulted in successful allo-HSCT after improvement of pulmonary function [8]. In addition, effective antiretroviral agents for HTLV-1 are essential for controlling both opportunistic infections and the neoplastic activity of ATL; however, they are not yet available. In conclusion, it is important to keep in mind that opportunistic infections can be a sign of HTLV-1 carrier or of smoldering ATL. Thus, HTLV-1 infection should be a differential diagnosis in patients with opportunistic infections such as CMV, PCP, or persistent herpes zoster and, if detected, correct staging of the ATL and appropriate management are necessary.

## Figures and Tables

**Table 1:** Laboratory data of two cases.

	Case 1	Case 2	
Year	2015	2010	2014
C-reactive protein (mg/dL)	5.00	0.17	5.07
WBC (/*µ*L)	15400	4700	2200
Hb (g/dL)	13.7	13.4	8.6
PLTs (/*µ*L)	604K	212K	149K
AST (U/mL)	20	23	33
ALT (U/mL)	28	19	35
LDH (122–228) U/mL	155	185	456
Total protein (g/dL)	5.9	7.8	4.8
Albumin (g/dL)	2.6	4.5	2.7
BUN (mg/dL)	13.3	7.4	10.5
Creatinine (mg/dL)	1.01	0.68	0.67
IgG (mg/dL)	1001	NT	NT
IgA (mg/dL)	249	NT	NT
IgM (mg/dL)	76	NT	NT
A/G ratio	NT	NT	1.58
Ca (8.7–10.3) mg/dL	8.7	9.4	8.4
sIL-2R (122–496) U/mL	4,304	293	14,058
HTLV-1 Ab (PA < ×16)	×1,024	×4,096	NT
HIV-Ab	Negative	Negative	Negative
HBV-Ag	Negative	Negative	Negative
HBV-Ab	Positive	Positive	Positive
HCV-Ab	Negative	Negative	Negative
CMV-IgM	Negative	Negative	Negative
CMV antigenemia (/50 × 10*e*3 WBC)	Positive (89, 91)	Negative (0)	NT
*β*-D-glucan (0–20) pg/mL	13.0	3.9	1,215
Pj-PCR in BAL (<4 × 10*e*1/*µ*g DNA)	NT	NT	9 × 10*e*5

Ag: antigen, Ab: antibody, PA: particle agglutination, NT: not tested, Pj: *Pneumocystis jirovecii*, and BAL: bronchoalveolar lavage.

**Table 2:** Flow cytometric data of peripheral blood mononuclear cells.

	Case 1 (%)	Case 2 (%)
CD2	92.2	99.2
CD3	81.0	91.5
CD4	83.8	87.1
CD5	90.3	95.5
CD7	34.8	14.9
CD8	6.7	7.6
CD4/CD8^*∗*^	12.5^*∗*^	11.5^*∗*^
CD10	0.2	0.2
CD19	7.0	0.4
CD20	7.0	0.5
CD23	4.0	0.7
Kappa	2.0	0.4
Lambda	1.7	0.4
CD11c	1.4	3.9
CD16	1.9	12.9
CD25	78.6	68.0
CD30	3.3	15.1
CD34	0.1	0.5
CD56	2.7	5.8
CD4+CD25+	75.5	87.2

^*∗*^Ratio.
